# An Ultrastretchable Electrical Switch Fiber with a Magnetic Liquid Metal Core for Remote Magnetic Actuation

**DOI:** 10.3390/polym13152407

**Published:** 2021-07-22

**Authors:** Kyeongmin Hong, Minjae Choe, Seoyeon Kim, Hye-Min Lee, Byung-Joo Kim, Sungjune Park

**Affiliations:** 1 Department of Polymer-Nano Science and Technology, Department of Nano Convergence Engineering Jeonbuk National University, Jeonju 54896, Korea; kmhong1@jbnu.ac.kr (K.H.); chzhgksk7@naver.com (M.C.); kseoy0126@gmail.com (S.K.); 2 R&D Division, Korea Institute of Carbon Convergence Technology, Jeonju 54853, Korea; leehm@kcarbon.or.kr; 3 Department of Carbon-Nanomaterials Engineering, Jeonju University, 303 Cheonjam-ro, Jeonju 55069, Korea; kimbyungjoo@jj.ac.kr

**Keywords:** stretchable elastic fiber, liquid metal, magnetic liquid metal composite, stretchable electronics

## Abstract

In this work we describe a soft and ultrastretchable fiber with a magnetic liquid metal (MLM) core for electrical switches used in remote magnetic actuation. MLM was prepared by removing the oxide layer on the liquid metal and subsequent mixing with magnetic iron particles. We used SEBS (poly[styrene-b-(ethylene-co-butylene)-b-styrene]) and silicone to prepare stretchable elastic fibers. Once hollow elastic fibers form, MLM was injected into the core of the fiber at ambient pressure. The fibers are soft (Young’s modulus of 1.6~4.4 MPa) and ultrastretchable (elongation at break of 600~5000%) while maintaining electrical conductivity and magnetic property due to the fluidic nature of the core. Magnetic strength of the fibers was characterized by measuring the maximum effective distance between the magnet and the fiber as a function of iron particle concentration in the MLM core and the polymeric shell. The MLM core facilitates the use of the fiber in electrical switches for remote magnetic actuation. This ultrastretchable and elastic fiber with MLM core can be used in soft robotics, and wearable and conformal electronics.

## 1. Introduction

Stretchable electronics have attracted enormous attention in the past decade due to their potential for application in soft robotics [1,2,3,4], bioelectronics [5,6,7], wearable [8,9,10] and conformal electronics [11,12,13,14]. Among the various conductors for creating the stretchable electronics, liquid metals, gallium-based alloys, are compelling because they are inherently stretchable while maintaining electrical conductivity [7,15]. Liquid metal spontaneously forms a thin passivating oxide layer (~3 nm) on the surface in the presence of oxygen, which promotes adhesion to various substrates [16]. Thus, it is possible to pattern liquid metals into desired geometries on the substrates via unique ways such as injection and vacuum-assisted filling into capillary networks and molding using elastomeric stamps [17,18,19].

Recently, liquid metal has been utilized to form a polymer-metal composite by dispersing the liquid metal particles into polymeric network [20,21,22,23,24,25,26]. Although the small particles of the liquid metal can prevent coalescence due to the thin oxide layer on the particles [26], the oxide layer can lead to poor dispersion of the metal in the polymer. The liquid metal composites with heterogeneous metals have demonstrated enhanced electrical, mechanical behavior and magnetic property, however, the oxide layer of the liquid metal also prevents direct contact between the liquid metal with the surroundings and interferes with the creation of the liquid metal composites preserving fluidic properties [16]. Thus, it is necessary to modify the oxide outer layer to obtain fluidic liquid metal-based composites [27,28,29].

Among the various liquid metal composites, magnetic liquid metal (MLM) is appealing because it possesses magnetic properties, electrical conductivity and remote actuation ability upon applying magnetic field [30,31]. Previously, MLM has been prepared by mechanical shear mixing of the liquid metal with magnetic particles [32,33] and doping of the liquid metal with iron particles cross-linked with the polymeric matrix [34]. The MLMs have demonstrated various actuations including three dimensional stretching in both horizontal and vertical direction [35], reversible splitting and merging by applying magnetic field [36,37], however, the magnetic particles interfere with the formation of the composites preserving fluidic behavior, thus actuating of the composites may be limited dynamically. It is therefore desirable to prepare the MLM composites preserving fluidic nature for application in soft and stretchable electronics that can actuate dynamically by applying magnetic field.

Previously, a hollow elastic fiber with liquid metal core prepared by injection of the liquid metal was presented [38,39,40]. The fiber is elastic, ultrastretchable [41,42,43] and maintains metallic conductivity due to nearly unlimited deformability of the liquid metal core. The fiber can have shape memory by utilizing phase transition of the liquid metal core from liquid to solid [39]. This ability can also enhance the toughness of the fiber [40]. Building on this prior study, we demonstrate ultrastretchable and elastic fiber with both electrical and magnetic properties by injecting the MLM into the fiber for use in electrical switch by magnetic actuation. The MLM can be prepared by removing the oxide layer of gallium and subsequent mixing with magnetic iron particles. The MLM exhibits fluidic behavior, thus it can be injected into the hollow elastic fiber at ambient pressure and maintains both magnetic and electrical conductivity even under high strain. We used the fiber with the MLM core in electrical switch by remote magnetic actuation.

## 2. Materials and Methods

### 2.1. Preparation of Magnetic Liquid Metal (MLM)

In order to prepare magnetic liquid metal, iron particles of 5~9 μm diameter (Sigma-Aldrich, St.Louis, MO, USA) 5, 10, 15, and 20 wt% were added to gallium (99.99%, Indium Corporation, Clinton, New York City, NY, USA) and treated with drops of diluted HCl (20 wt% in deionized water) to remove oxide layer on gallium. Subsequently, it was stirred with a glass rod for 2 min for complete mixing and then HCl was removed using a disposable pipette and a cotton swab. For convenience, the mass ratio of iron particles in gallium is presented using the Greek symbol φ.

### 2.2. Fabrication of Ultrastretchable and Elastic Hollow Fiber with MLM Core

We used two elastic fibers. SEBS (poly[styrene-b-(ethylene-co-butylene)-b-styrene]) triblock copolymer hollow fiber (Figure S1) was produced by conventional melt spinning a commercial thermoplastic elastomer (Kraton G1643, Kraton, Houston, TX, USA). To prepare magnetic elastic fiber, a silicone (Exsil-100, Gelest, Morrisville, PA, USA) composite was prepared by mixing the silicone with iron particles 10, 20, and 30 wt%. Once the composite was spun onto PET film, a stainless needle (21 G, outer diameter 0.81 mm) was rolled onto the film to be coated in the silicone composite and pick the needle up. Subsequently, the composite was cured at 100 °C for 4 h and peeled off from the needle to form a hollow fiber. The MLM wires with various iron particle concentrations were injected into the core of the hollow fiber at ambient pressure. We used the SEBS fiber with MLM core for electrical switch application due to benefit from the large scale process of the fiber. For convenience, the mass ratio of iron particles in silicone is presented using the Greek symbol ψ.

### 2.3. Characterization

The mechanical properties of the fibers were measured using an extensometer (Quasar 2.5 single column, Galdabini, Cardano al Campo, Italy) with a 1 kN load cell with a deformation rate of 3 mm min^−1^. To measure the magnetic strength of the fibers, a permanent magnet (NdFeB, 35 N grade, 50 × 10 × 5 mm^3^) was fixed under the top load cell of the extensometer, and placed a fiber measuring 5 cm in length at the bottom of the load cell. The magnet was approached to the fiber until the fiber was adhered to the magnet. We measured this maximum distance and calculated the mean value and standard deviation to define the maximum effective distance as the magnetic strength. The surface elements and topographies of liquid metal and magnetic liquid metal were characterized by scanning electron microscopy (SEM, SUPRA40VP SEM, Carl Zeiss, Oberkochen, Germany) and energy dispersive spectrometry (EDS, Ultim Max 100, Oxford Instruments, Abingdon, UK).

## 3. Results and Discussion

**Figure 1:**
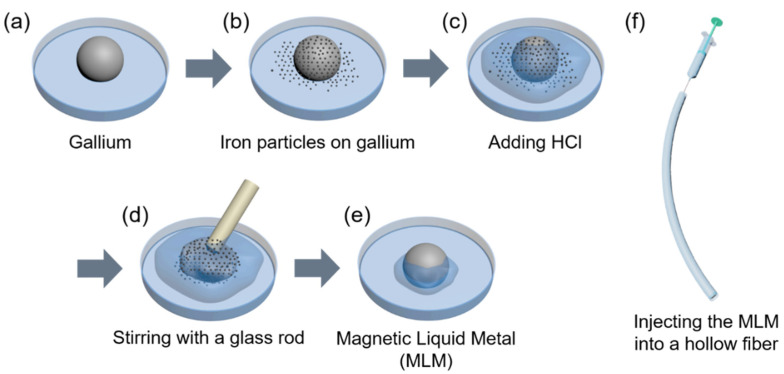
Preparation of the magnetic liquid metal (MLM) and its injection into a hollow fiber. (**a**) Gallium on a petri dish, (**b**) Gallium and iron particles on a petri dish, (**c**) Addition of HCl to immerse gallium and iron particles, (**d**) Stirring the mixture with a glass rod, (**e**) Magnetic liquid metal (MLM), (**f**) Injecting the MLM wire into a core of the hollow fiber.

Gallium spontaneously forms a native oxide layer on its surface, and thus it can be patterned into desired geometries on the substrates by various approaches. However, the oxide layer prevents gallium in promoting fluidic composite with different metals [16]. We also observed the adherence of iron particles on the surface of gallium due to the oxide layer and the particles were eventually trapped to form clusters. Thus, we prepared the MLM by removing the oxide layer of gallium in hydrochloric acid (HCl) and subsequent mixing with magnetic iron particles (Figure 1). The iron particles penetrate into gallium in the absence of oxide skin by galvanic replacement generated due to the standard reduction potentials of gallium (−0.560 V) lower than that of iron (−0.447 V) [35,44]. Once gallium is oxidized in HCl solution after oxide layer is removed, the iron particles supplied with electrons react with hydrogen ions to generate hydrogen gas [31,35,44]:

2Ga (s) − 6e^−^ → 2Ga^3+^ (aq) (1)

6H^+^ (aq) + 6e^−^ → 3H_2_ (g) (2)

Although the MLM composites exhibit the variable viscosity depending on the iron particle concentration, they preserve fluidic property when the iron particle concentration is less than 20 wt%, thus they can be injected into the capillary network of the hollow fiber at ambient pressure. The MLM wire in the fiber spontaneously forms the oxide layer that can prevent leakage of the MLM wire from the fiber.

**Figure 2:**
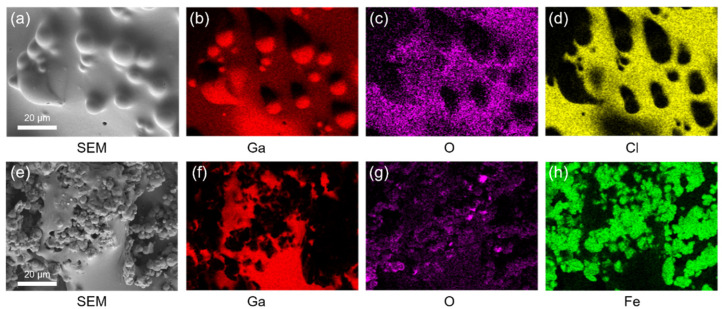
(**a**) SEM image and (**b**–**d**) Energy dispersive spectroscopy (EDS) element mapping images for (**b**) Ga, (**c**) O, (**d**) Cl on the surface of magnetic liquid metal prepared in the absence of oxide layer on gallium. (**e**) SEM image and (**f**–**h**) EDS element mapping images for (**f**) Ga, (**g**) O, (**h**) Fe on the surface of magnetic liquid metal prepared in the presence of oxide layer on gallium.

To investigate the effect of oxide layer on gallium for mixing with iron particles, two composites, i.e., the MLM formed in the absence of oxide layer and gallium mixed with iron particles in the presence of oxide layer, were characterized by SEM and elemental analysis via EDS. As shown in Figure 2a, the MLM with 10 wt% of iron particles (φ = 10%) showed surface bumps due to the alignment of iron particles on the surface along with the magnetic field. We note that iron particles are dispersed in the bulk of liquid metal homogeneously but aligned on the surface upon applying the magnetic field. The EDS analysis shows both gallium (Figure 2b) and oxygen (Figure 2c) elements on the MLM surface. The Cl signal is also visible because of the residual HCl solution in the MLM. No signal for iron particles was detected on the MLM surface, indicating that iron particles were dispersed beneath the gallium surface. Figure 2e–h shows the surface of gallium mixed with iron particles in the presence of oxide layer on gallium. The SEM image and EDS analysis shows aggregation of iron particles on the gallium surface, indicating the absence of penetration of iron particles into gallium due to the oxide layer.

**Figure 3:**
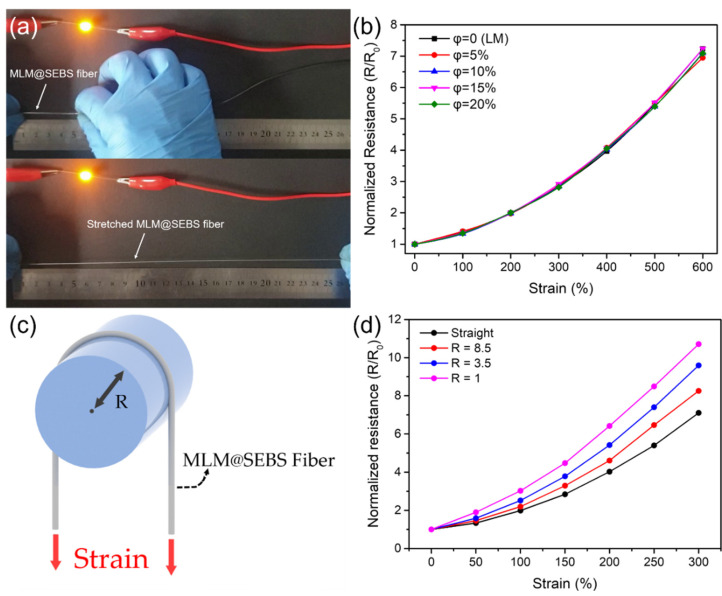
(**a**) A relaxed, 5 cm section of ultrastretchable SEBS fiber with the MLM core with 10 wt% of iron particles in the MLM wire (top), the fiber was stretched to more than 25 cm while maintaining conductivity (bottom). (**b**) The normalized resistance changes of the SEBS fiber with liquid metal core and the MLM core with iron particles with the concentration of 5, 10, 15, and 20 wt% (φ) as a function of strain. (**c**) Schematic showing the 180° bending tensile test. (**d**) The normalized resistance changes of the SEBS fiber with the MLM core with 10 wt% of iron particles in the MLM wire as a function of strain and bending radius (R).

As shown in Figure 3a, the SEBS fiber with the MLM core is ultrastretchable and exhibits electrical conductivity (Video S1). The MLM wires with various iron particle concentrations in the fibers exhibit the identical normalized resistance without strain due to metallic conductivity and the resistance of the MLM wires increases uniformly and linearly as a function of strain (Figure 3b) because of the geometric deformity in the MLM wire [38,45]. We note that the liquid metal with iron content less than 20 wt% can preserve fluidic property, thus it was possible to characterize the electrical behavior as a function of strain. The MLM wire in the silicone composite fiber, i.e., the silicone mixed with iron particles, shows electrical behavior identically, i.e., increased normalized resistance as a function of strain (Figure S2). This indicates that the electrical property of the liquid metal and the MLM wire is not affected by encapsulating materials.

To investigate the electrical behavior of the MLM wire affected by different deformation of the geometries, a tensile force was applied while the fiber with MLM core were bent on a rod. (Figure 3c). A 10 cm of the SEBS fiber with MLM core folded in half around a rod with various radius (R) is stretched to 300% (Figure 3c). The normalized electrical resistance of the MLM wires with various iron particle concentrations of 5, 10, 15, and 20 wt% increases as a function of strain (Figure S3). We also observed that the normalized resistance is slightly and inversely increased as a function of bending radius because small radius can highly deform the fiber (Figure 3d), i.e., generating larger deformation angle. The electrical conductivity of the MLM wire was maintained even while the fiber was highly deformed, i.e., in the knotted state (Figure S4 and Video S2).

**Figure 4:**
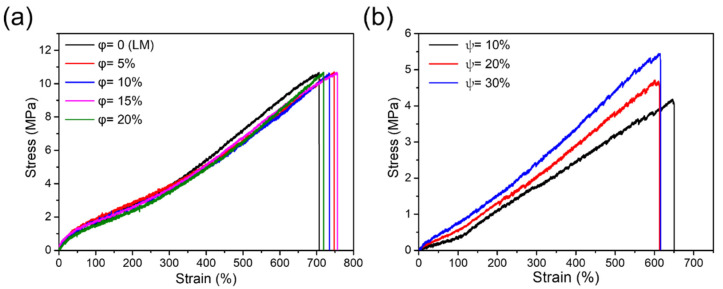
(**a**) Stress versus strain for the SEBS fibers with the MLM core as a function of iron particle concentration of 0, 5, 10, 15, and 20 wt% in the MLM wire (φ), (**b**) Stress versus strain for the hollow silicone (Exsil-100) composite fibers with MLM core as a function of iron particle concentration of 10, 20, and 30 wt% in the polymeric shell (ψ).

Figure 4a shows mechanical behavior of the hollow elastic SEBS fibers (the cross-section area was similar to the circle diameter of 1.4 mm) with the MLM core (the cross-section area similar to 0.4 mm diameter) as a function of iron particle concentration in the MLM wire. The fibers with the MLM wires with various iron particle concentrations exhibit identical tensile strength (~10.7 MPa), Young’s modulus (~4.4 MPa), and high elongation at break (>600% of elongation at break). These results indicate that the iron content in the liquid metal does not affect the fluidic property of gallium; however, MLM with more than 20 wt% of iron particles is highly viscous, and thus it is hard to inject into a fiber at ambient pressure. We also characterized mechanical properties of the silicone composite fibers with the liquid metal core. The fibers were fabricated by mixing a silicone (Exsil-100) with iron particles. Figure 4b shows the stress-strain curves of the silicone composite fibers with liquid metal core as a function of iron particle concentration in the polymeric shell. The composite fibers (1.85 mm diameter) with liquid metal core (0.8mm diameter) exhibit high stretchability (>600% of elongation at break) and low Young’s modulus. However, Young’s modulus (1.63~1.89 MPa) and tensile strength (3.8~5.4 MPa) of the fibers slightly increase as a function of iron particle concentration in the polymeric shell due to the rigid particle-induced stiffness.

**Figure 5:**
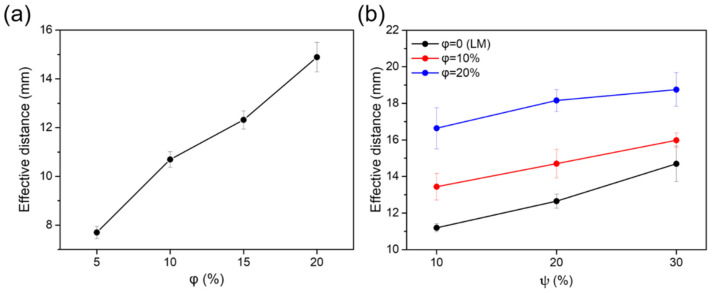
(**a**) The maximum effective distance between magnet and the hollow SEBS fiber with MLM core as a function of iron particle concentration of 5, 10, 15, and 20 wt% in the MLM core (φ). (**b**) The maximum effective distance between magnet and the hollow silicone composite fiber with the liquid metal (LM) core and MLM core as a function of iron particle concentration of 10, and 20 wt% in the MLM core (φ) and 10, 20, and 30 wt% in the polymeric shell (ψ).

The MLM wire in the fiber contains magnetic iron particles distributed in the liquid metal, thereby imparting magnetic properties to the wires. Thus, the fiber with MLM core can adhere to a magnet at certain distance which is defined as the maximum effective distance showing the magnetic strength. Figure 5a shows the magnetic strength of the hollow elastic fibers with the MLM cores as a function of the iron particle concentration (φ). The magnetic strength of the fiber is enhanced as a function of iron particle concentration in the MLM core. Figure 5b shows the magnetic strength of the hollow silicone composite fibers with liquid metal (LM) core and MLM core, respectively. The magnetic strength of the fibers is enhanced as a function of iron particle concentration in the polymeric network (ψ). It is also observed that the silicone composite fiber with MLM core exhibits the stronger magnetic strength than that of the fiber with LM core. Accordingly, the magnetic strength of the fiber can be enhanced by incorporating magnetic particles into the polymeric shell and the liquid metal core.

**Figure 6:**
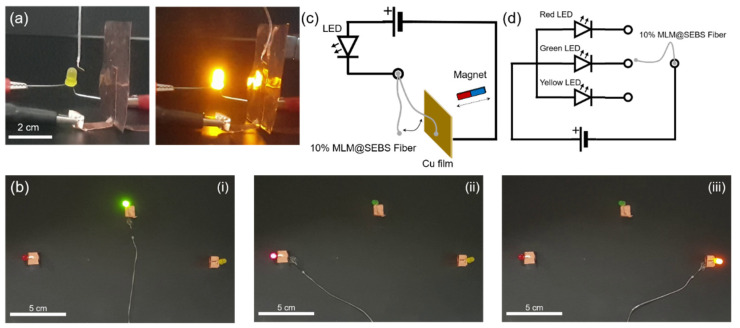
Electrical switching of a hollow elastic fiber with the MLM core containing 10 wt% of iron particles. (**a**) (left) Electrical circuit used for magnetic actuation of the SEBS fiber with the MLM core (MLM@SEBS fiber) before applying magnetic field, (right) LED activation by contacting the fiber with copper plate via remote magnetic actuation, (**b**) The fiber switched on (**i**) green LED, (**ii**) red LED, and (**iii**) yellow LED via remote magnetic actuation, (**c**,**d**) The electric circuit diagrams for (**a**,**b**), respectively.

Figure 6 shows the use of the SEBS fiber with the MLM core as an electrical switch to activate a light-emitting diode (LED) via remote magnetic actuation. The MLM core with 10 wt% of iron particles was used for demonstrating the application because of the moderate viscosity and magnetic property among the MLM composites with iron particles in the range of 5~20 wt%. [28] The MLM core is responsive to the magnetic field and thus enables the vertically suspended fiber to move toward the magnet behind copper electrode (Figure 6a,c). Once the fiber contacts the electrode via magnetic attraction, a LED was activated (Video S3). As shown in Figure 6d, the electric circuit is composed of three LEDs (red on the left, green in the center, and yellow on the right) and the SEBS fiber with the MLM core. Figure 6b depicts a switching demonstration of the fiber by applying magnetic field through a magnet under the paper. The green LED was switched on and then the fiber moved to switch on the red LED and the yellow LED (Video S4). The fiber with the MLM core is flexible, thus it can move by applying magnetic field and turn the LEDs on because of electrical conductivity of the MLM wire.

## 4. Conclusions

In this work, we have developed a facile approach to fabricate hollow elastic fibers with magnetic liquid metal cores for electrical switches via remote magnetic actuation. The fiber is stretchable while maintaining electrical conductivity due to the fluidic nature of the MLM core. The MLM prepared by removing the oxide skin of the liquid metal and subsequent mixing with magnetic particles exhibits both electric and magnetic responses. The MLM can be injected into the capillary network of the fiber at ambient pressure. The magnetic strength of the MLM wire and hollow elastic fiber increases as a function of the concentration of iron particles in the metal and polymeric shell. The MLM core facilitates the application of the fiber in electrical switch to open circuit and activate LEDs via remote magnetic actuation. This ultrastretchable elastic fiber with MLM core can potentially be used for application in electronic textiles, soft robotics, wearable devices, and stretchable magnetic actuators.

## Supplementary Materials

The following are available online at https://www.mdpi.com/article/10.3390/polym13152407/s1, Figure S1: Schematic of MLM@SEBS fiber and the chemical structure of (poly[styrene-b-(ethylene-co-butylene)-b-styrene]) triblock copolymer (SEBS) fiber, Figure S2: Normalized resistance changes of the LM@composite fiber and 5, 10, 15, and 20% MLM@composite fiber as a function of strain, Figure S3: Normalized resistance changes of the LM@SEBS fiber and 5, 10, 15, and 20% MLM@SEBS fiber as a function of strain and bending radius (R), (a) R = 8.5 mm, (b) R = 3.5 mm, and (c) R= 1 mm. Figure S4: Stretching of a knotted 10% MLM@SEBS fiber to 600% strain while maintaining conductivity. Video S1: Stretching of a 5 cm 10% MLM@SEBS fiber to 25 cm while maintaining conductivity. Video S2: Stretching of a knotted 10% MLM@SEBS fiber to 600% strain while maintaining conductivity. Video S3: Activation of a LED by contact with a MLM@SEBS fiber via remote magnetic actuation. Video S4: Switching three LEDs by contact with a MLM@SEBS fiber via remote magnetic actuation.
