# Supplementary material for: An Ultrastretchable Electrical Switch Fiber with a Magnetic Liquid Metal Core for Remote Magnetic Actuation
Source: Polymers (Basel). 2021 Jul 22;13(15):2407. doi: 10.3390/polym13152407 (PMC8348917; doi:10.3390/polym13152407)
Supplement: Supplementary file 1 [file polymers-13-02407-s001.zip › Supplementary materials_revised.pdf]

Supplementary Materials

Ultrastretchable Electrical Switch Fiber with Magnetic Liquid Metal Core for Remote Magnetic Actuation

Kyeongmin Hong [1], Minjae Choe [1], Seoyeon Kim [1], Hye-min Lee [2], Byung-Joo Kim [3] and Sungjune Park [1,*]

[1] Department of Polymer-Nano Science and Technology; Department of Nano Convergence Engineering, Jeonbuk National University, Jeonju, 54896, Korea

[2] R&D Division, Korea Institute of Carbon Convergence Technology, Jeonju 54853, Korea

[3] Department of Carbon-Nanomaterials Engineering, Jeonju University, 303 Cheonjam-ro, Jeonju 55069, Korea

E-mail: s.park@jbnu.ac.kr

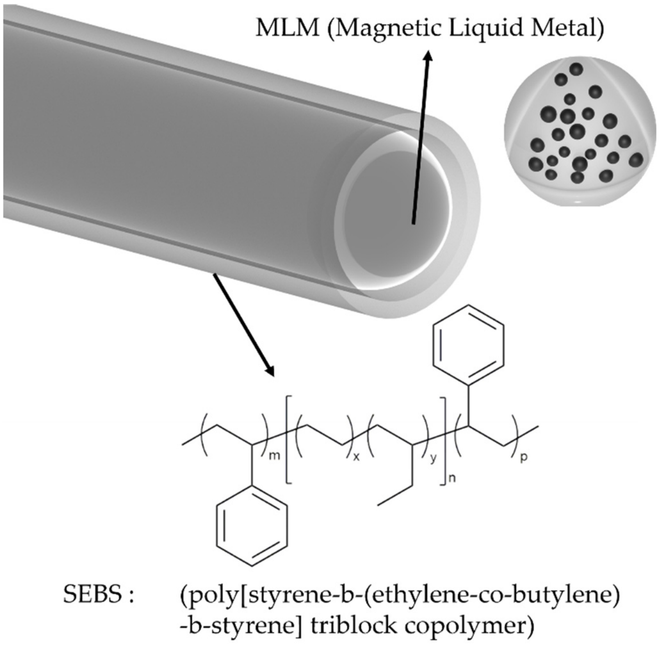

Figure S1. Schematic of MLM@SEBS fiber and the chemical structure of poly[styrene-b-(ethylene-co-butylene)-b-styrene]) triblock copolymer (SEBS) fiber.

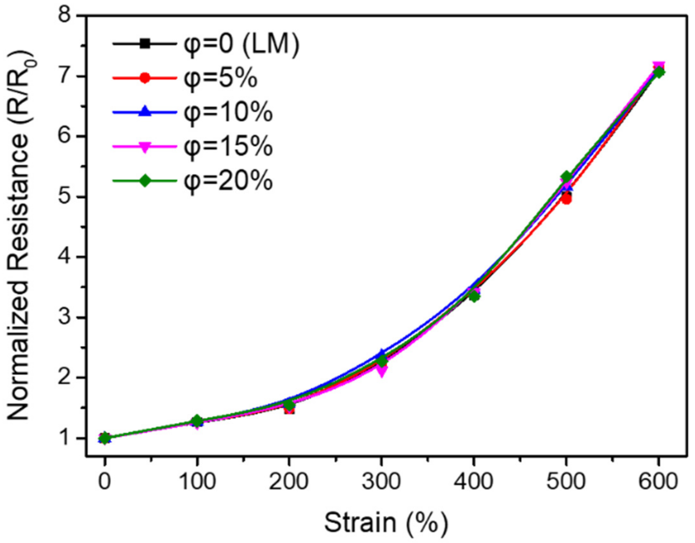

Figure S2. Normalized resistance changes of the LM@composite fiber and 5, 10, 15, and 20% MLM@composite fiber as a function of strain.

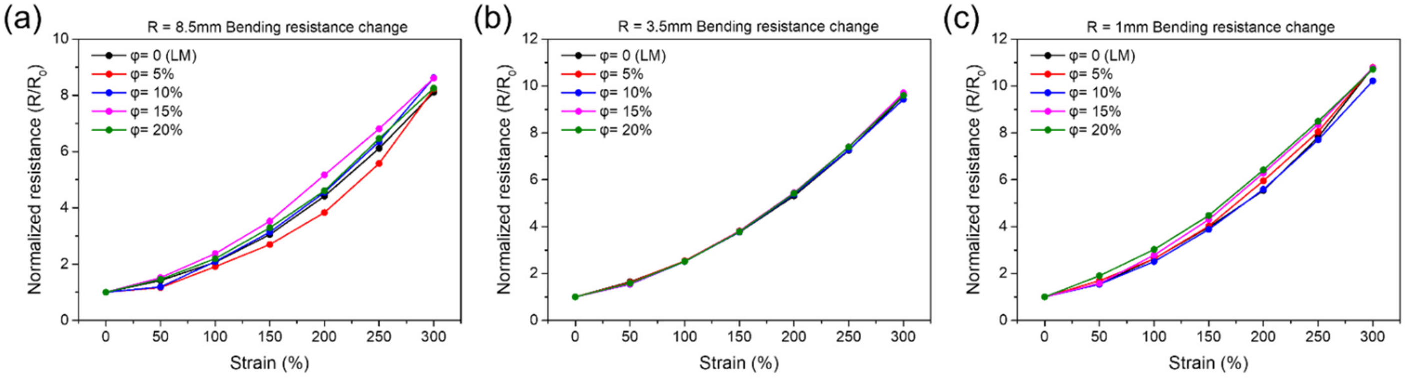

Figure S3. Normalized resistance changes of the LM@SEBS fiber and 5, 10, 15, and 20% MLM@SEBS fiber as a function of strain and bending radius (R), (a) R= 8.5 mm, (b) R= 3.5 mm, and (c) R= 1 mm.

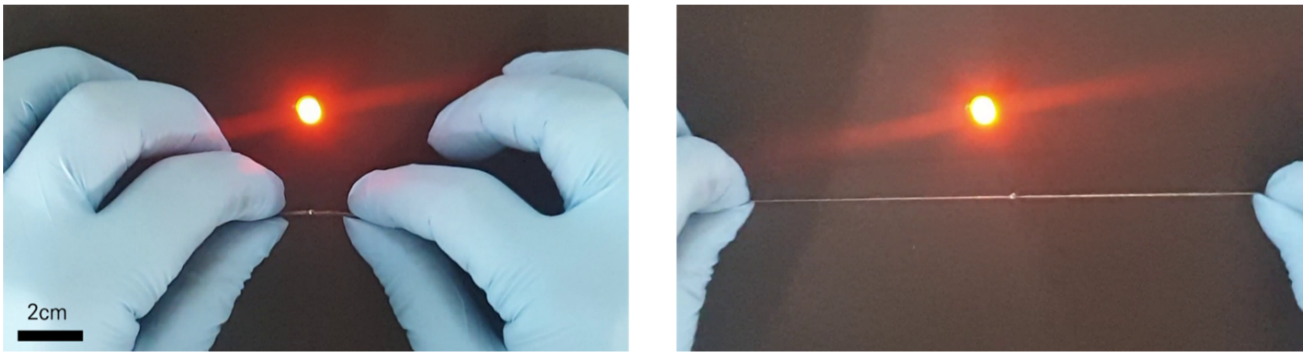

Figure S4. Stretching of a knotted 10% MLM@SEBS fiber to 600% strain while maintaining conductivity.

Supplementary Video List

Video S1. Stretching of a 5 cm 10% MLM@SEBS fiber to 25 cm while maintaining conductivity.

Video S2. Stretching of a knotted 10% MLM@SEBS fiber to 600% strain while maintaining conductivity.

Video S3. Activation of a LED by contact with a MLM@SEBS fiber via remote magnetic actuation.

Video S4. Switching three LEDs by contact with a MLM@SEBS fiber via remote magnetic actuation.
